# Frameless Stereotactic Insertion of Viewsite Brain Access System with Microscope-Mounted Tracking Device for Resection of Deep Brain Lesions: Technical Report

**DOI:** 10.7759/cureus.1012

**Published:** 2017-02-04

**Authors:** Tim White, Shamik Chakraborty, Rohan Lall, Andrew A Fanous, John Boockvar, David J Langer

**Affiliations:** 1 Department of Neurosurgery, Hofstra Northwell School of Medicine; 2 Brain Tumor Center, Department of Neurosurgery, Hofstra Northwell School of Medicine; 3 Brian Tumor Center, Department of Neurosurgery, Hofstra Northwell School of Medicine; 4 Neurosurgery, State University of New York at Buffalo

**Keywords:** deep brain lesions, viewsite brain access system, stereotactic neurosurgery

## Abstract

The surgical management of deep brain tumors is often challenging due to the limitations of stereotactic needle biopsies and the morbidity associated with transcortical approaches. We present a novel microscopic navigational technique utilizing the Viewsite Brain Access System (VBAS) (Vycor Medical, Boca Raton, FL, USA) for resection of a deep parietal periventricular high-grade glioma as well as another glioma and a cavernoma with no related morbidity. The approach utilized a navigational tracker mounted on a microscope, which was set to the desired trajectory and depth. It allowed gentle continuous insertion of the VBAS directly to a deep lesion under continuous microscopic visualization, increasing safety by obviating the need to look up from the microscope and thus avoiding loss of trajectory. This technique has broad value for the resection of a variety of deep brain lesions.

## Introduction

Surgical access to deep targets is a common challenge in neurosurgery. Stereotactic needle biopsies often provide insufficient tissue or result in a sampling bias, potentially leading to a misdiagnosis, especially in the case of smaller lesions [1-2]. In addition, they do not allow for complete surgical resection. Transcortical approaches permit better sampling and resection, but are associated with the morbidity related to larger and longer operations, as well as cortical resection [3]. Additionally, certain lesions are not anatomically amenable to transcortical approaches or neuroendoscopy.

The use of both neuronavigation and microscopes is commonplace in these cases. However, their combination is often serial in nature, requiring surgeons to toggle back and forth between technologies [4]. Neuronavigation can guide an approach, and microsurgery can ensure minimal morbidity, but currently their integration is often limited.

An important surgical principle when accessing deep brain pathology is maximizing accuracy and minimizing brain injury. Some models have been recently proposed in order to enhance neuronavigation and targeting of deep brain lesions. Intraoperative magnetic resonance imaging (MRI) and ultrasound guidance have demonstrated improved surgical outcomes [5-9]. Beyond the use of imaging alone, Oppenlander, et al. suggested the use of robotic auto-positioning to improve targeting and the visualization of deep brain lesions by developing an algorithm combining the microscope with the neuronavigation [10]. However, all of these techniques have drawbacks such as the availability and the lack of proven cost effectiveness of intraoperative MRI, the inability of auto-positioning to account for objects in the field, collision of the microscope with objects in the operating room, and increased cost [11-12].

Another new technique is the Viewsite Brain Access System (VBAS) (Vycor Medical, Boca Raton, FL, USA), which allows for access to deep lesions with minimal impact on the surrounding tissues [13]. The plastic cylindrical retractor employed in this technique provides access while minimizing morbidity by gently splitting rather than resecting white matter.

In order to better target deep brain lesions, we combined the VBAS, neuronavigation, and microsurgery in a manner that allowed for an accurate approach along with minimal disturbance of normal parenchyma under constant microscopic visualization. Attaching the universal tracker of the Stryker Cranial Navigation System (Stryker, Kalamazoo, MI, USA) to the microscope facilitates the use of the latter as a navigational tool. Importantly, the Leica microscope (Leica Microsystems, Wetzlar, Germany) has a lock capability, allowing the surgeon to fixate on a single location thus defining a trajectory for the approach. Once locked in place, the depth of focus on the microscope can be changed to target lesions at various depths. A VBAS length may be subsequently chosen in order to address the depth of the lesion. By advancing the VBAS into the brain under microscopy, keeping the crosshairs of the microscope centered and in focus, access to a deep brain lesion can be achieved with maximal accuracy and minimal disruption of the surrounding parenchyma.

## Technical report

### Clinical presentation

A 59-year-old male, with a history of an arteriovenous malformation (AVM) that was surgically resected in 1998, presented to Lenox Hill Hospital with worsening seizures as well as speech and gait disturbance. A physical exam on admission was significant for right-sided tremor, dysmetria, and some right-sided weakness.

The patient underwent an MRI scan that revealed a left posterior parietal periventricular ring-enhancing lesion, measuring 1.7 cm x 1.0 cm. Perfusion MRI demonstrated hyperperfusion. Fluid-attenuated inversion recovery (FLAIR) sequences showed signal abnormality, which extended throughout the left frontal, parietal, and temporal white matter and into the corpus callosum to the contralateral right parietal periventricular white matter. Positron emission tomography–computed tomography (PET-CT) using fluorodeoxyglucose (FDG) showed increased FDG activity in the left posterior frontal lobe periventricular white matter, which corresponded to the location of the lesion.

After discussing the imaging findings with the patient and the family, both opted for an excisional biopsy of the deep brain lesion.

### Operative technique

The patient was brought to the operating room and was placed in the lateral position on a beanbag with the right arm held in a sling. The head was subsequently positioned in the Mizuho Sugita Frame (Mizuho America, CA, USA). Registration of fiducial markers on the patient and the frame were then completed with the Stryker Cranial Navigation System. An 8 cm elongated S-shaped skin incision was then made to the level of the bone overlying the left parietal calvarium. A standard parietal craniotomy was performed, exposing the superior parietal lobule.

**Figure 1:**
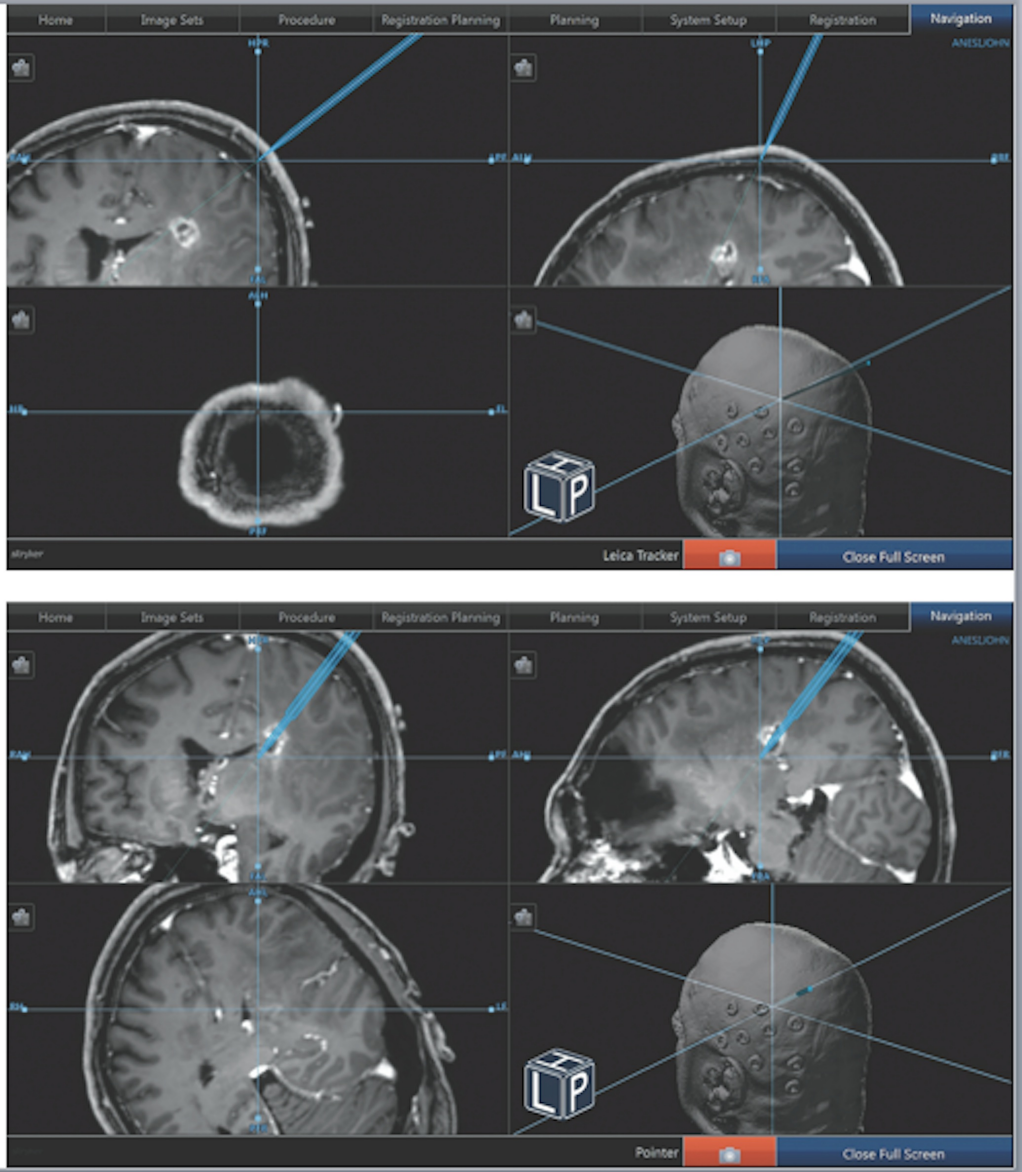
Advancement of the VBAS via Neuronavigation Using the “tool” setting on the neuronavigation system, we were able to mimic the depth of the VBAS retractor system by changing the focus of the microscope. The microscope was fixed in place and the Stryker neuronavigation setup was turned to the “tool” setting. A) Shows the initial placement of the tool, where the microscope was set to its most shallow focus. B) As the focus of the microscope was focused deeper toward the lesion, the tool pedicle on the neuronavigation software increased its depth. By maintaining the distal most portion of the VBAS retractor at the maximal focus point of the microscope, the surgeon was able to track the depth of the retractor as it related to the stereotactic navigation.

**Figure 2:**
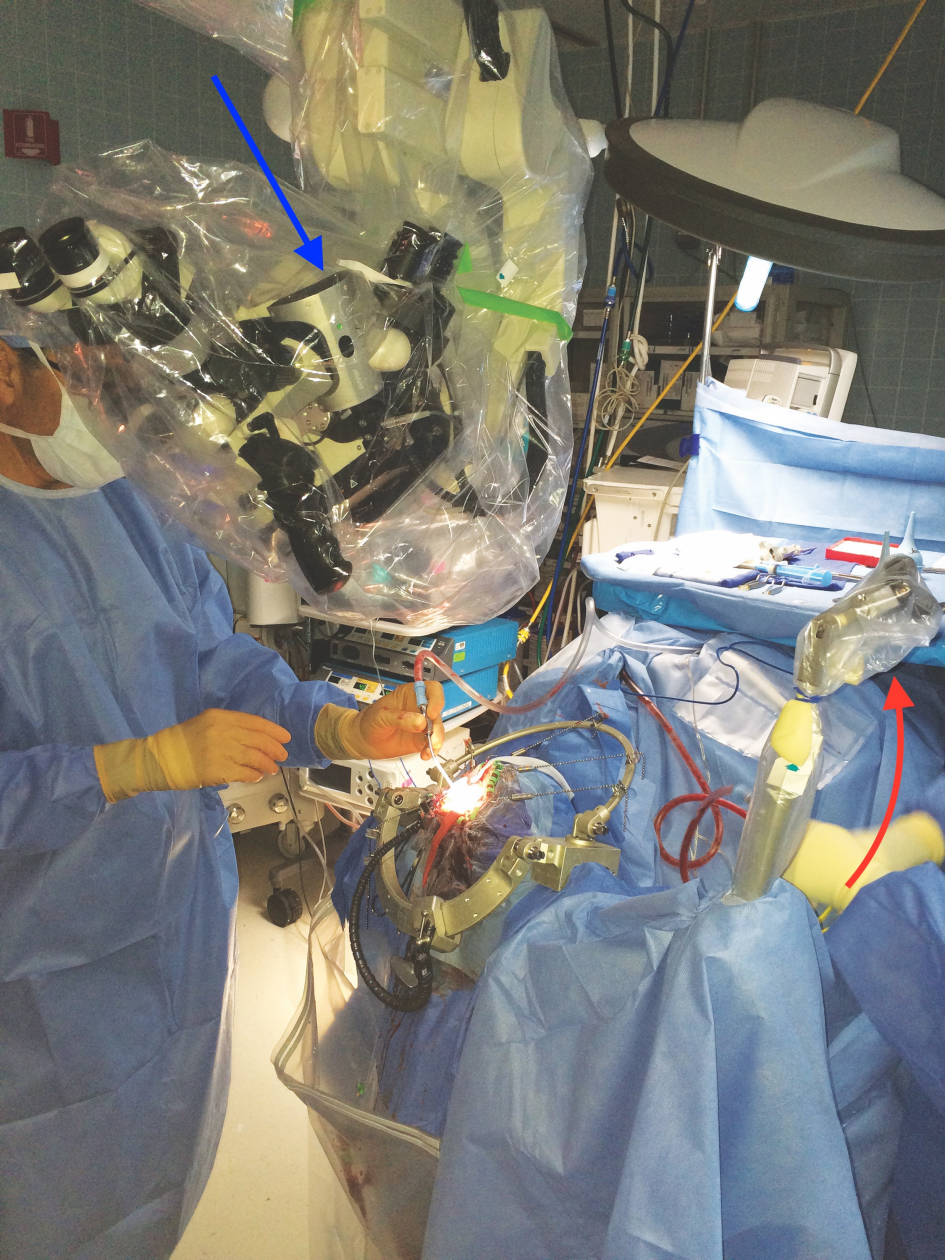
Operative Field This figure shows the set up of the operative field. Here, the microscope is fixed in place with the mounted tracker attached (seen at the arrow). Fixing the microscope allowed for the use of the microscope as an extension of the neuronavigational tools. The crosshairs of the microscope are focused on the center of the VBAS retractor, and as the VBAS is advanced, the focus is altered, thereby altering the depth of the tool on the neuronavigation screen. The blue arrow shows the scope-mounted tracker. The red arrow shows the frame-mounted tracker.

The Leica microscope with the attached Stryker Cranial Navigation System was then registered to the position. The microscope was subsequently moved into place over the superior parietal lobule. At this point, a surgical trajectory that would permit comfortable surgical resection was planned, and the microscope was fixed in place (Figures 1-2).

**Figure 3:**
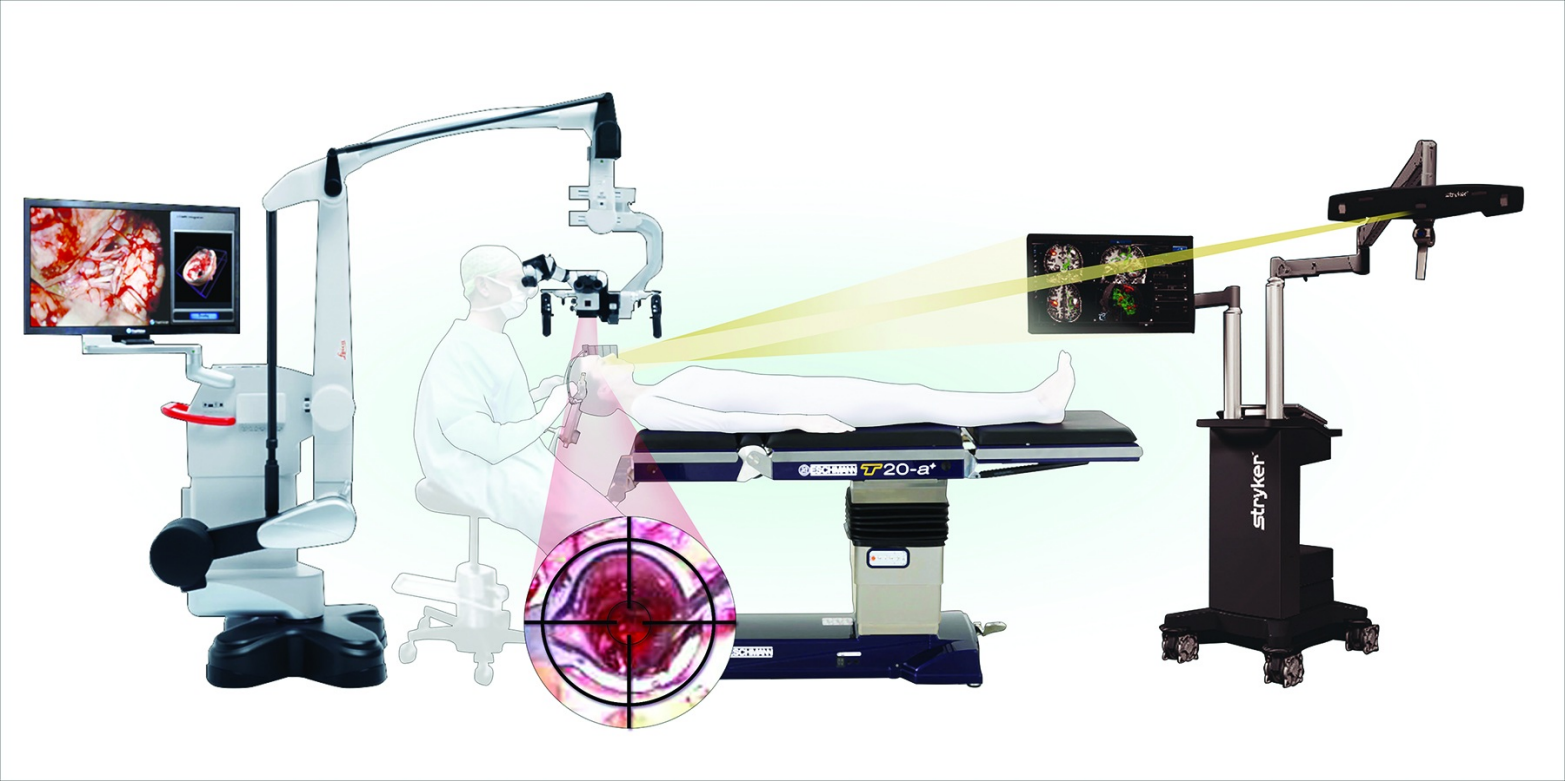
Operating Room Set Up This figure is an illustration of the entire operative set up. Here, the surgeon is positioned with the fixed microscope viewing the VBAS retractor, as centered in the crosshairs of the microscope. As the focus of the microscope increases its depth, the surgeon advances the VBAS retractor to follow the “tool” icon on the neuronavigation software.

The crosshairs as seen through the eyepieces of the fixed microscope were then used to target the deep lesion on the neuronavigation (Figure 3).

The scope was not used during the initial targeting procedure.

The surface of the brain was coagulated and a small incision made. The VBAS was then placed in line with the crosshairs of the microscope, over the superior parietal lobule corticectomy. A 5 cm VBAS was chosen based on the depth of the lesion in order to give at least 2 cm of clearance about the parenchyma. The VBAS was then carefully inserted into the brain and advanced to the lesion. The tubular and tapered nature of the VBAS allowed for the smooth, blunt dissection of the cortex with minimal disruption. Once at the desired depth, the inner stylet of the VBAS was removed. Once the inner stylet was removed, the distal end was opened, allowing for visualization of the surgical field. The direction of the retractor may be adjusted during the procedure by simply changing the angle of the retractor. The depth may be altered as well, by re-introducing the stylet for further advancement. At no point did the surgeon need to remove their eyes from the surgical field to check the navigation for proper trajectory and was able to visualize the full course of the insertion of the VBAS (Figure 2). The surgeon did not remove his eyes from the microscope.

**Figure 4:**
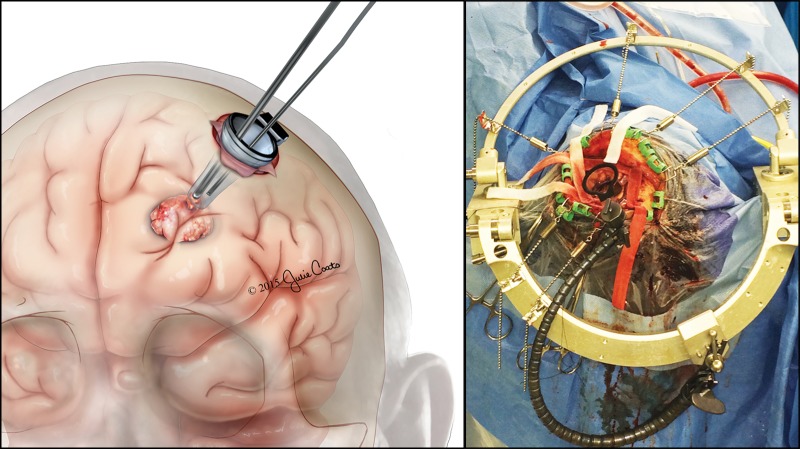
Orientation and Insertion of the VBAS Retractor This figure shows a view of the operative field. A) An illustration of the insertion of the VBAS retractor and the localization of the deep lesion. B) Shows the Sugita frame with the VBAS cylindrical retractor stabilized in place. The length of the VBAS retractor was chosen based on the known depth of the lesion. Here the retractor is advanced to the depth of the lesion.

Once properly positioned, the retractor was firmly affixed to a Layla arm on the Sugita frame (Figure 4).

The lesion was then resected using a standard microsurgical technique after the frozen section revealed a high-grade glioma in the eloquent cortex. The patient was extubated and remained in a stable neurological condition. Pathological analysis was consistent with high-grade glioma, and postoperative imaging demonstrated the absence of hematoma with a typical postoperative resection cavity.

### Further illustrative cases

Case 2

A 49-year-old female presented with a three-month history of left hand numbness and paresthesia. A physical exam was significant for transient left hemianesthesia. MRI / magnetic resonance (MR) angiogram showed a right posterior thalamic paraventricular cavernoma extending into the ventricular atrium and measuring 2.8 cm x 3.0 cm. Following discussions with the patient and family, the decision was made to proceed with surgical resection.

The patient’s head was fixed in the lateral position in the Sugita frame and the Stryker Navigation System was registered. An incision was made over the right parietotemporal region. The temporalis muscle was partially divided, and two burr holes were performed followed by a parietal craniotomy. The dura was then opened in a cruciate fashion. Utilizing the VBAS with the Stryker Navigation System linked to a Leica microscope via a tracker, an inferior parietal lobule trajectory to the lesion was used. A corticectomy was performed and we inserted a 4.5 cm depth Vycor tube (Corning, NY, USA) directly to the lesion under constant microscopic visualization. Once positioned, the retractor was firmly affixed to a Layla arm on the Sugita frame.

Upon initial dissection, the atrium of the right lateral ventricle was entered, but with further dissection, the mass was localized. The leakage of cerebrospinal fluid (CSF) from the ventricle resulted in minimal brain shift. The cavernoma was able to be localized with a minimal adjustment of the retractor system. Using suction and bipolar, the cavernoma was dissected, and the lesion as well as the hemosiderin stained tissue was removed. Once this was completed, hemostasis was achieved. The VBAS was then removed and hemostasis was achieved through the entry tract. Once the tube was removed, transcortical signals were performed that demonstrated baseline signals; however, as a result of the transcortical neuromonitoring, a generalized seizure occurred. The field was flooded with cold irrigation, and Propofol was bolused by the anesthesia team. This intervention broke the seizure, but did put the patient into burst suppression for the next several minutes of the case. Once the patient was stabilized and hemostasis was achieved, the dura was closed, the craniotomy flap was fixed into place, the galea was sutured, and the skin was closed. The patient had no neurological complications postoperatively. Postoperative MR disclosed expected postoperative change with apparent total resection of the cavernoma.

Case 3

A 25-year-old woman presented with a two-year history of left neck and facial spasms, as well as migraines. Physical exam was significant for bilateral clonus as well as brisk bilateral deep tendon reflexes. MRI scans were performed twice, separated by 10 months. The first set of imaging demonstrated a small lesion of the left cerebellar hemisphere near the vermis with moderate compression of the posterior left midbrain. The lesion did not enhance on the first set of imaging, but did enhance on the more recent MRI. Following discussions with the patient and the family, both opted for surgical resection.

With the help of stereotactic navigation, a linear incision was made from the inion to the top of C2. A suboccipital craniotomy was subsequently performed. The Leica microscope with the attached Stryker Cranial Navigation System were then registered to the position, with a plan for a supracerebellar approach. At this point, a trajectory was planned and the microscope was fixed in place with the crosshairs of the microscope fixed to target the lesion. The VBAS was then placed in line with the crosshairs after a small incision of the pia mater was made. The VBAS was subsequently advanced within the crosshairs, and the focus of the microscope was adjusted as the VBAS depth was changed. The lesion was encountered after blunt dissection. The retractor was left without firm attachment to a retractor arm to permit a full range of motion during resection. A grayish circumscribed lesion was delineated from the brainstem. Gross total resection was achieved, and pathological analysis confirmed the diagnosis of pilocytic astrocytoma WHO I. The patient had no neurological complications postoperatively.

## Discussion

The operative microscope revolutionized the field of neurosurgery, allowing surgeons to better visualize and manipulate fine structures. Since the introduction of the microscope to the field of neurosurgery by Theodore Kurze in 1957, its functionality has been ever increasing [5]. Recently, steps have been taken to expand microsurgery by uniting microscopes with neuronavigation and intraoperative imaging [7].

Neuronavigation has also become a ubiquitous part of a neurosurgical practice [6]. The principle components of frameless stereotactic surgery include a probe, a computer-based image module, and a reference. Frameless stereotactic navigation has been shown to achieve accuracy of 2–3 mm based on electrode-based studies, as well as good accuracy and safety regarding access of deep intracranial lesions [11,14-15]. Errors in frameless stereotaxy generally involve factors outside the computer-based modules, such as the quality / timing of the preoperative imaging or factors that cause brain shift during the surgery [16].

The use of neuronavigation is invaluable in the resection of brain tumors. Retrospective data suggests that aggressive resection of glioblastoma and other glial tumors results in improved overall survival. For instance, a threshold of 78% of resection was shown to improve the outcome in patients with glioblastoma, and the extent of resection was shown to be a positive predictor of outcome in patients with glioma [17-18]. Microsurgical resection remains the standard treatment for newly diagnosed glioblastoma, followed by radiation and chemotherapy. Improved outcomes were seen in both complete and subtotal resections [18].

The VBAS allows for cortical retraction using a clear tubular introducer and a channel of variable widths and depths, producing a gentle splitting of cerebral parenchyma that minimizes damage to the cerebral tissue. Retractor width and length can be chosen prior to the operation based on the navigational depth and size of the targeted lesion. Once the lesion is encountered intraoperatively, the retractor can then be pivoted, allowing for complete debulking and increased access. The VBAS carries the advantage of providing a white matter splitting approach with a built-in retractor system. Initial studies have shown promising results by employing this retractor system to approach deep brain lesions. For instance, one series of nine patients showed evidence of white matter damage using T2 weighted fluid attenuated inversion recovery (T2/FLAIR) in only one patient who subsequently had no clinical complications [19]. Other studies have also suggested that this technology can be useful in the targeting of deep brain pathology other than brain tumors [20].

However, this technique is not without its shortcomings. The VBAS comes in multiple lengths and width variations, but with deeper lesions, the maneuverability of instruments may be decreased due to the rigid retractor system. Also, ideally, there should be a method to link microscope focus to the retractor to automatically maintain focus as the retractor is advanced. Of note, one complication not seen in this series may be the presence of retractor tract hemorrhage. As the introducer and retractor bluntly dissect, there is no source of hemostasis but for the pressure from the retractor system. If hemorrhage was encountered, this would likely require the withdrawal of the retractor system and the continuation of the procedure without the VBAS.

Here, we present the concurrent registration of the surgical microscope to the neuronavigation system combined with the VBAS to permit continuous visualization during the insertion of the retractor in a predetermined trajectory (Figure 1). The advantage of using this technique is improved safety, in that the clear VBAS and the close-up view under the microscope allows the surgeon to avoid injury to critical structures. At the same time, by fixing the trajectory of the microscope and advancing the VBAS, the surgeon does not have to look away from the field, limiting the chances of deviating away from the proper trajectory. We believe this simple and elegant technique can improve patient safety and outcomes, as well as permit superior diagnosis and resection of intracranial lesions.

## Conclusions

We present a novel technique for the resection of deep brain lesions. This is the first described combination of a microscope-mounted tracker guided by frameless stereotactic navigation, which was used to position a VBAS retracting system into a deep brain location to effectively excise a deep brain lesion. This operative technique illustrates a viable and enhanced approach to deep brain lesions with minimal associated morbidity.
